# The impact of resveratrol on the outcome of the in vitro fertilization: an exploratory randomized placebo-controlled trial

**DOI:** 10.1186/s13048-024-01391-7

**Published:** 2024-04-15

**Authors:** A. Conforti, G. G. Iorio, R. Di Girolamo, M. Y. Rovetto, S. Picarelli, F. Cariati, R. Gentile, A. D’Amato, O. Gliozheni, B. Fioretti, C. Alviggi

**Affiliations:** 1 Department of Neuroscience, Reproductive Science and Odontostomatology, School of Medicine, University of Naples “Federico II, Naples, Italy Via Sergio Pansini, 5, 80131; 2 Department of Public Health. School of Medicine, University of Naples “Federico II, Naples, Italy; 3 Department of Chemistry, Biology and Biotechnologies, University of Perugia, Perugia, Italy; 4 Department of Interdisciplinary Medicine, University of Bari, 1St Unit of Obstetrics and Gynecology, Bari, Italy; 5 Head of Department of Obstetrics and Gynecology, University Hospital for Obstetrics & Gynecology, University of Medicine of Tirana, KocoGliozheni”, Tirana, Albania

**Keywords:** Ovarian sensitivity, FORT, FOI, FSH, POSEIDON, Hypo-response

## Abstract

**Background:**

Resveratrol is a natural polyphenolic compound present in plants and red wine with many potential health benefits. This compound has various anti-inflammatory and anti-tumor properties and can improve cellular mitochondrial activity. This trial was designed to evaluate the effect on the outcome of IVF of Resveratrol supplementation in women > 35 years with good ovarian reserve (AMH > 1.2 ng/ml). Women were randomized to receive or placebo or Resveratrol (150 mg per day) for three months preceding the ovarian stimulation (OS). All patients were stimulated with a starting dose of recombinant FSH ranging between 150 and 300 IU according to age and ovarian reserve. GnRH antagonist flexible protocol was adopted for pituitary suppression. Triggering was performed with urinary hCG (10.000 IU).

**Results:**

The study was conducted between January 2019 and December 2022 with aa total of 37 cases and 33 controls were recruited. No statistically significant differences in the number of oocytes retrieved, biochemical pregnancy, clinical pregnancy and live birth rates were observed between women treated with resveratrol and control group. A statistically significant increase in the follicle output rate (FORT) and follicle-to oocyte index (FOI) was observed in women treated with resveratrol-based nutraceutical (0.92 *versus* 0.77 [*p* = 0.02], and 0.77 *versus* 0.64 [*p* = 0.006], respectively).

**Conclusions:**

Preliminary results from this study indicate that pre-treatment with resveratrol may improve ovarian sensitivity to exogenous FSH, which in turn may decrease the risk of hypo-response to OS in advanced reproductive age women.

## Introduction

Resveratrol is a natural polyphenolic compound present in plants and red wine with numerous health properties. Several in vivo and in vitro studies have demonstrated that this compound has various anti-inflammatory and anti-tumor properties [1, 2]. Moreover, resveratrol can improve cellular mitochondrial activity and trigger a series of molecular mediators capable of counteracting some of the most important metabolic mechanisms underlying aging [3]. Mitochondria are major regulators of multiple vital cellular processes, including apoptosis, calcium homeostasis, and adenosine triphosphate (ATP) generation through the metabolic pathway known as oxidative phosphorylation [4]. A significant reduction in mitochondrial DNA levels has been reported in female oocytes in advanced maternal age hypothesizing a fundamental role in embryonic development and in vitro fertilization (IVF) success [5]. Nonetheless, the effect of resveratrol on the ovarian sensitivity is still unknow. The ovarian sensitivity reflects the ability of the ovaries to response properly to exogenous gonadotropin. Women characterized by a reduced ovarian sensitivity (or “hypo-response”) are characterized by reduced the number of eggs retrieved despite the normal ovarian reserve markers. Evidence indicates that hypo-response is typically observed in women with reduced IVF prognosis [6–10]. So far, several ovarian sensitivity indicators have been proposed [11]. The main indicators of ovarian sensitivity are summarized in Table 1.

**Table 1:** Main indicators of ovarian sensitivity reported in literature

Ovarian sensitivity indicators	How to calculate
**Follicle Output RaTe (FORT)** [12, 13]	Ratio between number of pre-ovulatory follicles obtained after COS and the number of antral follicles at the beginning of OS
**Follicle-to Oocytes Index (FOI)** [11, 14]	Ratio between the number of retrieved oocytes and the number of antral follicles at the beginning of OS
**Ovarian Sensitivity Index (OSI)** [15]	Ratio between number of oocytes retrieved and total dose of gonadotrophins administered

Considering the relevance of ovarian response and aging on IVF success, the aim of the present exploratory randomized placebo-controlled trial was to evaluate the effect of resveratrol-based supplementation on ovarian responsiveness in advanced reproductive age women with normal ovarian reserve markers candidates to IVF.

## Methods

### Study design

This randomized, single-blind, controlled, single-center, experimental study enrolled infertile women attending in vitro fertilization (IVF) at the IVF Unit of University Federico II, Naples, Italy, from January 2019 and December 2022. The study was approved by the local Ethics Committee of the University of Naples Federico II on January 2019 (n.328/18).

The study was conducted in accordance with the Declaration of Helsinki and Good Clinical Practice guideline [16]. This study was reported fulfilling POSORT and CONSORT recommendations [17, 18]. The study followed the guidelines on infertility testing that emerged from the Harbin Conference [19]. Patients fulfilling the inclusion and exclusion criteria (Table 2) were invited to participate in the study [20–23]; the study was explained to all participants. Both oral informed consent and written informed consent were obtained by all patients included.

**Table 2:** Study protocol selection criteria

INCLUSION CRITERIA	EXCLUSION CRITERIA
Age ≥ 35 years	Presence of ovarian neoformations at the time of OS
AMH > 1.2 ng/ml [20]	Body Mass Index (BMI) < 18 and > 28 kg/m^2^
Regular menstrual cycle (every 25–35 days)	Polycystic ovary syndrome [21]
Indication for IVF/ICSI: idiopathic, tubal factor, mild and moderate male factor, maternal age	Endocrinopathies (disorders of the hypothalamic-pituitary axis, dysthyroidism or hyperprolactinaemia)
No previous ovarian surgery	Endometriosis III-IV Stage [22]
	Diabetes type I e II
	Severe male factor [23]

### Randomization

We employed a stratified randomization scheme together with permuted block randomization. Randomization is then performed using PASS software with package blockrand for each stratum at a 1:1 allocation ratio and with block size determined as 2, 4, 6 and 8. The randomization list was prepared by an independent statistician not participating in the recruitment. Sequentially numbered, opaque sealed envelopes are used for allocation. Envelopes receive numbers in advance and are opened sequentially, only after the participant’s name has been written on the appropriate envelope. One member of the research team who labelled the containers was aware of the allocation. Researchers were unaware of treatment allocation until the envelope is opened. Treatment was not masked to care providers and investigators but masked to the participants and the outcome assessor.

### Clinical and ultrasonographic evaluation

For each patient we collected: age, BMI Kg/m^2^, menstrual cycle characteristics, last menstrual period, basal FSH and LH, ovarian reserve markers (AMH and AFC) and number of years of infertility. The ultrasound examinations were executed using a Voluson E8 device (GE Healthcare, Zipf, Austria) with a transvaginal probe and they were all performed by two sonographers (G.G.I and SP) within the fifth day of OS. AFC was assessed using 2D-ultrasound and 3D-volume to reduce interobserver variability [24]. AFC was assessed considering 2–10 mm antral follicles using trans-vaginal pelvic ultrasound (multi-frequency vaginal probe 5.0–7.5 MHz) according to the most recent guideline [25].

### Intervention

Patients of study group received 3 months of treatment, before undergoing OS, with a nutraceutical formulation (2 daily capsules) containing *trans*-resveratrol and a form of resveratrol supported on Magnesium dihydroxide (total amount of resveratrol 150 mg) [26, 27], folic acid (400 mcg), vitamin D (25 mcg), vitamin B12 (2.5 mcg), and vitamin B6 (1.4 mg) (GENANTE®, S&R Farmaceutici, Bastia Umbra PG, Italy), while control group received 3 months of treatment with placebo, containing only excipient (microcrystalline cellulose, vegetable Magnesium, Stearate (E470b), croscaramellose sodium (E468), talc (E553b), Silicon Dioxide (E551), Arabic gum (E414) and no active ingredients. The 150 mg/day dose is not based on dose-finding studies. The dose is dependent on the recommendation of European Food Safety Authority (EFSA), which considers 150 mg daily the maximum safe dose for the human population without restrictions [28]. The pretreatment duration of 3 months before IVF was arbitrary, as no studies were available at the time of the study design and motivated by the duration of the transition from pre-antral to mature follicles taking approximately 80–90 days [29]. After completing the treatment, patients undergo OS, oocyte retrieval and fresh embryo transfer.

Patients of both groups performed OS as follows:

- on day 2–3 of the menstrual cycle recombinant FSH (r-FSH) were administrated based on age and ovarian reserve (from 150 to 300 IU daily);
- all patients were treated with flexible GnRH antagonist protocol at a dose of 0.25 mg/day in case of dominant follicle at the ultrasound (mean diameter > 13 mm or estradiol levels > 300 pg/ml);
- in women with at least two follicles of mean diameter > 17 mm, 10.000 IU of hCG or triptorelin 0.2 mg were administered as ovulation inducer, the latter was preferred in the presence of more than 20 follicles during ovarian stimulation, recovery of more than 24 oocytes, and estradiol levels above 3,500 pg/mL [30];
- oocyte retrieval (PU) was performed by ultrasound-guided transvaginal aspiration 34–36 h after the trigger;
- ultrasound-guided Embryo transfer (ET) was performed within 5 days of oocyte yield;
- the luteal phase, except for cases deemed to be at risk of hyper-response, was supported with 400 mg of micronized vaginal progesterone per day.

### Outcomes

The primary endpoint was the clinical pregnancy rate (CPR) per aspirated cycle (a pregnancy diagnosed by ultrasonographic visualization of one or more gestational sacs or definitive clinical signs of pregnancy). Secondary endpoints were major efficacy and efficiency endpoints in IVF: number of oocytes retrieved and number of mature oocytes (metaphase II oocytes), mature oocytes percentage, number of cleavage embryos and blastocysts, number of cryopreserved embryos, blastulation rate (for those patients underwent transfer on day five), defined as the percentage of inseminated oocytes reaching blastocyst stage, ovarian sensitivity indicators (FORT, FOI and OSI), estradiol (E_2_) levels at peak, duration of stimulation, total dose of gonadotropins, biochemical pregnancy rate (BPR) per aspirated cycle (a pregnancy diagnosed only by the detection of beta hCG in serum or urine), live birth rate (LBR) per aspirated cycle (delivery after 22 completed weeks of gestational age) [31].

### Statistical methods

Reproductive outcomes were compared between the resveratrol treated group (Study Group) and Control group. Continuous variables are expressed in terms of mean ± SD or median and interquartile range for parametric and non-parametric data, respectively. Categorical variables are expressed in terms of frequency and percentage. The distribution of continuous variables was evaluated with the Shapiro test. The two-sided t-test for independent samples was used to assess inter-group differences concerning parametric data. The two-sided Mann–Whitney U test was used to test inter-group differences for non-parametric data, whereas the Chi-square test was adopted to verify differences in terms of categorical data between groups. Results were analyzed using the statistical package SPSS 22 for Windows (Statistical Package for the Social Sciences, IBM, New York). A *p*-value < 0.05 was considered statistically significant.

### Sample size

Since no data were available in the literature at the time of the enrollment, the present study is to be considered an exploratory randomized trial, considering a total sample size of 100 patients (50 per group).

### Interim-analysis

In November 2022, an unplanned interim analysis was conducted on the basis of data from all randomly assigned patients. Results indicated a probability of more than 95% that we would find no significant differences in terms of CPR or LBR if we would include more patients in the trial. After considering all the evidence, the research group decided early closure of the trial.

## Results

**Fig. 1:**
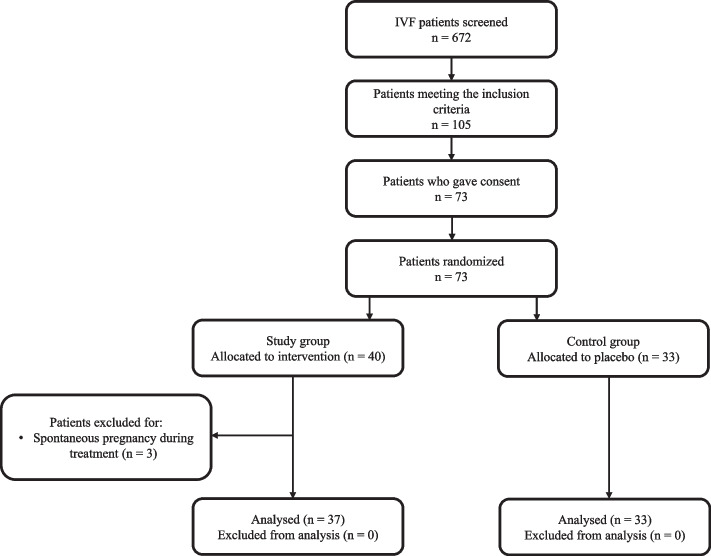
CONSORT flow diagram

A total of 73 women underwent randomization. Therefore, 40 patients were assigned to the test group and 33 to the reference group (Fig. 1).

Three patients in the study group conceived naturally over the 3 months of therapy and were excluded from the final analysis. Demographic and infertility history data are summarized in Table 3.

**Table 3:** General characteristics of enrolled women

	**Study Group *n* = 37**	**Control Group *n* = 33**	***p*-value**
AGE (years)	38.66 ± 2.18	38.04 ± 2.38	0.26
BMI (Kg/m^2^)	22.94 ± 2.95	22.86 ± 2.57	0.9
AFC	9.74 ± 3.8	10.3 ± 4.07	0.55
AMH (ng/ml)	1.95 (1.52–3.2)	2.15 (1.4–3.67)	0.91
FSH (IU/ml)	6.7 (6.15–7.55)	7.65 (6.15–9)	0.25
LH (IU/ml)	5.8 (3.87–7.42)	5.3 (4.25–6.25)	0.95
Years of sterility	4 (3–6.5)	3 (2-4)	**0.01**

Parametric continuous data are presented as mean ± standard deviation

Non parametric continuous data are presented as median and interquartile range

The mean age and the BMI of the women enrolled in the study were comparable in the two groups. The two groups were also comparable regarding ovarian reserve (AMH and AFC), basal FSH and LH. Infertility duration was higher in study group (*p* < 0.01). IVF outcomes are summarized in Table 4. All patients were triggered with 10,000 UI hCG.

**Table 4:** Ovarian stimulations outcomes, embryos and pregnancies between the two study groups

	**Study Group *n* = 37**	**Control Group *n* = 33**	***p*-value**
Total gonadotropin dose (IU)	1973.61 ± 395.14	1907.89 ± 475.08	0.53
E_2_ at peak (pg/ml)	1301 (996–2329.5)	1247 (901.5–1836)	0.53
Retrieved oocytes	7.89 ± 3.62	6.4 ± 3.84	0.1
M2 oocytes	6.26 ± 2.28	5.15 ± 2.7	0.1
Mature oocytes	0.89 (0.73–1)	0.88 (0.71–1)	0.76
FORT	0.92 (0.84–1)	0.77 (0.65–0.95)	**0.02**
FOI	0.77 (0.7–0.95)	0.64 (0.49–0.76)	**0.006**
OSI	4.44 (2.34–5.04)	2.86 (1.67–4.48)	0.25
Cleavage embryos/patient	2.16 ± 0.9	1.95 ± 1.14	0.39
Blastocysts/patient	1.47 ± 1.22	1 ± 1.21	0.11
Embryos transferred day 3	14/41 (34%)	15/39 (38.5%)	0.69
Embryos transferred day 5	23/41 (56%)	18/39 (46%)	0.37
Cryopreserved embryos/patient	0.79 ± 0.92	0.65 ± 0.93	0.53
Blastulation rate	0.3 (0.25–0.33)	0.25 (0.17–0.33)	0.45
BPR	9 (24.32%)	10 (30.3%)	0.57
CPR	9 (24.32%)	10 (30.3%)	0.57
LBR	6 (16.22%)	10 (30.3%)	0.16

*FORT* Follicle output rate, *FOI* Follicle oocytes indexm, *OSI* Ovarian sensitivity index, *BPR* Biochemical pregnancy rate, *CPR* Clinical pregnancy rate, *LBR* Live-birth rate

Parametric continuous data are presented as mean ± standard deviation

Non parametric continuous data are presented as median and interquartile range

Categorical variables are expressed in terms of frequency and percentage

The two groups were not significantly different in terms of pregnancy outcomes (BPR, CPR and LBR). The two groups were also similar in terms of mean number of collected oocytes and mature oocytes, percentage of mature oocytes, OSI, total gonadotropin dose and peak estradiol levels. FOI and FORT were significantly higher in the study group than in the control group (*p* < 0.02 and *p* < 0.006, respectively). Furthermore, no significant difference was observed in terms of embryos and blastocyst collected and blastulation rate.

## Discussion

In this trial resveratrol pretreatment for 3 months before IVF, while not associated with statistically significant differences in clinical pregnancy rate (primary endpoint), increases ovarian sensitivity to exogenous gonadotrophins in women undergoing OS. Indeed, a significantly increase in FORT and FOI was observed in study versus control group. Consistently an increased, despite not significant, OSI was observed in the study group compared with control group. To the best of our knowledge this is the first time that an effect of ovarian sensitivity is reported after resveratrol pretreatment before OS.

The mechanism by which the resveratrol could increase ovarian sensitivity should be still elucidated, despite several hypothesis could be proposed. The main one might be related to the positive effect exerted by resveratrol on mitochondrial activity. Resveratrol can increase mitochondrial mass in human granulosa cells (GC) through a mechanism involving reduction of voltage-dependent potassium currents, intracellular calcium homeostasis, and regulation of mitomiRNAs [32, 33]. Women with reduced ovarian responsiveness exhibit reduced mitochondrial mass, cholesterol uptake capacity and expression of enzymes involved in steroidogenesis, such as StAR (Steroidogenic Acute Regulatory), 3-beta-hydroxysteroid dehydrogenase (3-beta-HSD) and aromatase, compared with normal responders [34]. Low expression of these enzymes leads to a reduced estrogen and progesterone production even after OS. Since low responsiveness to FSH may correlate with reduced mitochondrial mass, we propose that the enhanced ovarian responsiveness observed in the present study is related to the ability of resveratrol to stimulate mitochondrial biogenesis in GC. Another mechanism of action could be related to the anti-inflammatory and antioxidant properties of resveratrol. Indeed, resveratrol might contrast the negative effect exerted by pro-inflammatory environmental factors related to the hypo-response physiopathology [35]. The possibility of improving the sensitivity of the ovary to gonadotropins could be useful in reducing the number of patients with an unpredictable hypo-response to OS, as in the case of POSEIDON groups 1 and 2 patients [12]. POSEIDON’s groups 1 and 2 encompass women who had poor (< 4) or suboptimal [4–9] number of oocytes retrieved after a conventional OS despite the presence of an adequate ovarian reserve, defined by an AFC of ≥ 5 and/or an AMH ≥ 1.2 ng/mL. Indeed, retrieval of fewer than 10 oocytes is associated with decreased cumulative live birth rates (CLBR) [36]. Thus, given a patient who fits POSEIDON’s groups 1 or 2, the final goal would be to find ways to maximize oocyte yield aiming at obtaining more than 9 oocytes at the end of stimulation [36, 37].

Other studies that have investigated the role of resveratrol in IVF are summarized in Table 5.

**Table 5:** Published protocols about resveratrol in IVF setting

Authors	Setting	Conclusion
Ochiai et al*.*, 2019 [38]	Cross-sectional retrospective study comparing the outcomes of embryo transfer cycles in women receiving resveratrol supplementation (200 mg/day) continuously with a control group	Resveratrol supplementation during embryo transfer cycles appears to be detrimental for pregnancy outcomes
Bahramrezaie et al*.*, 2019 [39]	Randomized controlled trial comparing the IVF outcomes in PCOS women receiving resveratrol supplementation (800 mg/day) continuously for 40 days before the initiation of ovarian stimulation with a control group	The number of mature oocytes, cleavage rate, fertilization rate, and fertility rate were not significantly different between the two groups, but resveratrol supplementation is associated high-quality oocyte rate and high quality embryo rate
Gerli et al*.*, 2021 [40]	Randomized controlled trial comparing the IVF outcomes in women receiving resveratrol supplementation (300 mg/day) continuously for 3 months before the initiation of ovarian stimulation with a control group	Resveratrol supplementation is associated with significantly higher numbers of oocytes and MII oocytes, higher fertilization rates, and higher numbers of embryos and blastocytes per patient. No significant differences in biochemical or clinical pregnancy, live birth, and miscarriage rates are revealed

A recently published randomized trial [40] reported a statistically significant increase in the number of oocytes retrieved in women pretreated with resveratrol. Despite higher oocytes yield in the study group, our data failed to find statistically significant differences in comparison with the controls. The discrepancy between the two trials could be related to both study populations and sample size. More specifically, our trial investigated the impact of resveratrol in advanced maternal age, which is associated per se with higher risk of suboptimal or poor ovarian response [41]. Indeed, small sample size may explain the reason why, at least in our series, the statistically significant increase in FORT and FOI was not reflected in a significantly higher oocyte yield, however a trend to more retrieved oocytes among the study group (*p* = 0,1) is shown.

In contrast with a retrospective analysis of Ochiai et al. we do not observe a detrimental effect of resveratrol on pregnancy outcome [38]. This could be probably due to the retrospective design, heterogenous population and different IVF protocol adopted in Ochiai study comparing with our trial (mild versus conventional stimulation). Furthermore, the amount of resveratrol prescribed in Ochiai et al. study (200 mg daily) was higher comparing with our trial (150 mg daily). Finally, it should be considered that the age at the oocyte retrieval in Ochiai et al. study was significantly higher in women who were supplemented with resveratrol comparing with control group [38].

The strengths of our study reside in the design, the prospective nature and the selective inclusion criteria involving specifically women with the worst IVF prognosis namely those with advanced reproductive age [42].

The limitation of our study lies in the low sample size. This makes it impossible to speculate on the comparison between the groups in terms of oocytes parameters (es. retrieved and blastocytes) and pregnancy outcomes. Should we have reached a higher enrollment number the positive trend might have been significant in accordance with a previous study Gerli. et al. [41]. Difficulties in achieving the planned sample size were mainly related to the COVID-19 pandemic.

At the time of enrollment, there was no published study on IVF outcomes in patients treated with resveratrol, so a sample size calculation could not be performed. Considering the differences observed in terms of clinical pregnancy rate in our study (24,3% vs 30,3%) a post hoc analysis revealed that we need 865 women per group with an alfa error set at 0.05 and power set 0.80. Nonetheless, the number of women recruited were adequate to detect a difference in terms of FOI and FORT with a power over 90%.

A further concern is that the population selected for the study (women older than 35 years and with good ovarian reserve markers) is the one most likely to benefit from taking the resveratrol-based nutraceutical, and therefore the data should be evaluated in other groups of patients.

The unpublished and ongoing RCT on resveratrol supplementation in IVF available on Clinicaltrials.gov are shown in Table 6.

**Table 6:** Unpublished RCT about resveratrol in IVF setting

Identification number	Title
NCT01782911	Effect of Resveratrol on Metabolic Parameters and Oocyte Quality in PCOS Patients Undergoing IVF Treatment
NCT06235294	Effects of Resveratrol Supplementation on Oocyte Quality in Advanced Maternal Aged Women Undergoing in Vitro Fertilization

In conclusion, despite sample size does not allow to address the impact of resveratrol prior OS on clinical pregnancy rate, the preliminary results of this study suggest that such a treatment improves ovarian sensitivity to exogenous FSH at least in women above 35 years of age. Following confirmation of our data, pre-treatment with resveratrol may decrease the risk of unexpected hypo-response to OS in advanced reproductive age women.

## Data Availability

Not applicable.
